# Circulating mRNAs are differentially expressed in pregnancies with severe placental insufficiency and at high risk of stillbirth

**DOI:** 10.1186/s12916-020-01605-x

**Published:** 2020-05-22

**Authors:** Natalie J. Hannan, Owen Stock, Rebecca Spencer, Clare Whitehead, Anna L. David, Katie Groom, Scott Petersen, Amanda Henry, Joanne M. Said, Sean Seeho, Stefan C. Kane, Lavinia Gordon, Sally Beard, Kantaraja Chindera, Smita Karegodar, Richard Hiscock, Natasha Pritchard, Tu’uhevaha J. Kaitu’u-Lino, Susan P. Walker, Stephen Tong

**Affiliations:** 1 Translational Obstetrics Group, Department of Obstetrics and Gynaecology, University of Melbourne, Mercy Hospital for Women, Level 4, Studley Rd, Heidelberg, Victoria 3084 Australia; 2 Mercy Perinatal, Mercy Hospital for Women, Heidelberg, Victoria 3084 Australia; 3 Department of Obstetrics and Gynaecology, University of Melbourne, Parkville, Victoria 3010 Australia; 4 Elizabeth Garrett Anderson Institute for Women’s Health, University College London, London, WC1E 6BT UK; 5 University of Leeds, Leeds, LS2 9JT UK; 6 Department of Maternal Fetal Medicine, Royal Women’s Hospital, Parkville, Victoria 3052 Australia; 7 Liggins Institute, University of Auckland, Auckland, 1023 New Zealand; 8 Centre for Maternal Fetal Medicine, Mater Mothers’ Hospital, South Brisbane, Queensland 4101 Australia; 9 School of Women’s and Children’s Health, UNSW Medicine, University of New South Wales, Sydney, Australia; 10 Maternal Fetal Medicine, Joan Kirner Women’s & Children’s Sunshine Hospital, St Albans, Victoria 3021 Australia; 11 The University of Sydney Northern Clinical School, Women and Babies Research, St Leonards, New South Wales 2065 Australia; 12 University of Melbourne Centre for Cancer Research, Parkville, Victoria 3010 Australia; 13 Department of anesthesia, Mercy Hospital for Women, Heidelberg, Victoria 3084 Australia

**Keywords:** Circulating mRNA, Pregnancy, Fetal growth restriction, Fetal hypoxia

## Abstract

**Background:**

Fetuses affected by placental insufficiency do not receive adequate nutrients and oxygenation, become growth restricted and acidemic, and can demise. Preterm fetal growth restriction is a severe form of placental insufficiency with a high risk of stillbirth. We set out to identify maternal circulating mRNA transcripts that are differentially expressed in preterm pregnancies complicated by very severe placental insufficiency, in utero fetal acidemia, and are at very high risk of stillbirth.

**Methods:**

We performed a cohort study across six hospitals in Australia and New Zealand, prospectively collecting blood from 128 pregnancies complicated by preterm fetal growth restriction (delivery < 34 weeks’ gestation) and 42 controls. RNA-sequencing was done on all samples to discover circulating mRNAs associated with preterm fetal growth restriction and fetal acidemia in utero. We used RT-PCR to validate the associations between five lead candidate biomarkers of placental insufficiency in an independent cohort from Europe (46 with preterm fetal growth restriction) and in a third cohort of pregnancies ending in stillbirth.

**Results:**

In the Australia and New Zealand cohort, we identified five mRNAs that were highly differentially expressed among pregnancies with preterm fetal growth restriction: *NR4A2*, *EMP1*, *PGM5*, *SKIL*, and *UGT2B1*. Combining three yielded an area under the receiver operative curve (AUC) of 0.95. Circulating *NR4A2* and *RCBTB2* in the maternal blood were dysregulated in the presence of fetal acidemia in utero. We validated the association between preterm fetal growth restriction and circulating *EMP1*, *NR4A2*, and *PGM5* mRNA in a cohort from Europe. Combining *EMP1* and *PGM5* identified fetal growth restriction with an AUC of 0.92. Several of these genes were differentially expressed in the presence of ultrasound parameters that reflect placental insufficiency. Circulating *NR4A2*, *EMP1*, and *RCBTB2* mRNA were differentially regulated in another cohort destined for stillbirth, compared to ongoing pregnancies. *EMP1* mRNA appeared to have the most consistent association with placental insufficiency in all cohorts.

**Conclusions:**

Measuring circulating mRNA offers potential as a test to identify pregnancies with severe placental insufficiency and at very high risk of stillbirth. Circulating mRNA *EMP1* may be promising as a biomarker of severe placental insufficiency.

## Background

Preterm fetal growth restriction arises when there is poor placental function at a preterm gestation. While preterm fetal growth restriction (such as requiring delivery prior to 34 weeks’ gestation) is uncommon, affecting 0.2–0.5% of pregnancies, it is one of the most severe complications of pregnancy. These fetuses are at high risk of stillbirth [1] or major perinatal injury that leads to permanent disability [2].

A slowing of growth that leads to preterm fetal growth restriction occurs because the under-functioning placenta cannot meet the nutrient and oxygen demands of the fetus for normal growth velocity. If severe, it can progress to fetal hypoxia and acidemia. The acidemia arises from an accumulation of lactic acid, produced because the fetus switches to anaerobic metabolism in the face of hypoxia. The lactic acid can cause asphyxial neuronal and myocardial injury, culminating in stillbirth.

Given there are no therapies to rescue in utero placental insufficiency [3], the current clinical management for preterm fetal growth restriction is to leave fetuses in utero to gain gestation, but to deliver promptly if there is evidence that the fetus is significantly acidemic. Ultrasound-based tests or the cardiotocograph (to detect ominous fetal heart rate patterns) are commonly used to detect the presence of pre-terminal fetal hypoxia/acidemia [4]. These capture physiological changes that are associated with fetal hypoxia or acidemia [5] such as increased resistance to blood flow in the uterine artery (maternal compartment) or the umbilical artery (placental compartment) [6], decreased resistance in the fetal middle cerebral artery (fetal compartment) [7], or the presence of specific fetal heart rate patterns [8].

While these tests have improved outcomes including perinatal survival, none of these existing tests can reliably determine which pregnancies are affected by significant fetal acidemia [8]. Even with the clinical availability of these tests, preterm growth-restricted fetuses are still lost to stillbirth or suffer significant perinatal asphyxial injury [9]. We therefore need better clinical tests to identify the presence of severe placental insufficiency and to flag fetuses that are extremely compromised and at imminent risk of stillbirth, or severe injury in utero leading to permanent disability after birth.

Detecting biochemical/molecular changes in the presence of significant placental insufficiency and fetal hypoxia may be a new strategy to monitor fetal wellbeing in these high-risk pregnancies and could add precision to existing clinical tests. RNAs are shed from many organs, including the placenta [10], and released into the circulation where they can be sampled and measured [11, 12]. Furthermore, there are numerous reports demonstrating differential expression in the circulation of RNAs coding various genes when there is significant pathology, such as cancers [13, 14].

Circulating RNA concentrations of some genes vary with such predictability across pregnancy that their maternal signature might be able to determine gestational age with an accuracy comparable to ultrasound fetal measurements [15]. It is also possible that circulating RNAs could be used as novel clinical biomarkers of obstetric disease. Most studies have explored their use in the diagnosis or prediction of pregnancy complications such as preeclampsia [16, 17] and spontaneous preterm birth [15].

We propose a conceptually different use of circulating RNAs: to monitor fetal wellbeing by identifying fetuses with severe placental insufficiency and fetal acidemia that are at high risk of stillbirth or neonatal morbidity. Beside our small pilot study [18], the concept of measuring circulating RNAs to monitor fetuses at risk of stillbirth has not been explored.

Here we report a large multi-center cohort study where maternal blood obtained just prior to delivery was used to identify circulating RNAs that were associated with preterm fetal growth restriction, fetal acidemia, and stillbirth. We did mass RNA seq in one cohort, but not all three cohorts. Hence the line, as originall written, could be slightly misleading. differentially expressed with disease. We then validated genes that were differentially expressed in an independent cohort from a different continent and in a third cohort of pregnancies that ended in stillbirth from severe preterm fetal growth restriction.

## Methods

### Study participants for our cohorts

#### Fetal Oxygenation (FOX) Study

We collected maternal blood samples from 128 women with preterm growth-restricted fetuses.

Participants were recruited across five tertiary hospitals in Australia: Mercy Hospital for Women, Heidelberg (in Victoria); Royal Women’s Hospital, Parkville (in Victoria); Sunshine Hospital, St Albans (in Victoria); Mater Mothers Hospital, South Brisbane (Queensland); Royal Hospital for Women, Randwick (New South Wales); and Royal North Shore Hospital, St Leonards (New South Wales). We also collected samples from a sixth site, the National Women’s Health Auckland City Hospital, Grafton, in New Zealand. Ethics approval was obtained from all institutions (approval numbers: MHW R11/04, RWH + Sunshine Hospital 10/41, MMH 1928 M, RHW 12/240, RNSH 1305-151 M, NWH, ACH 12/NTA/96/AM02), and all women provided written informed consent.

Preterm fetal growth restriction was defined as a customized birthweight < 10th centile (www.gestation.net, Australian parameters) requiring iatrogenic delivery prior to 34 weeks’ gestation with uteroplacental insufficiency (asymmetrical growth + abnormal artery Doppler velocimetry +/− oligohydramnios +/− abnormal fetal vessels velocimetry). We excluded fetal growth restriction due to infection, chromosomal or congenital abnormalities, and multiple pregnancy.

All blood samples were collected within 2 h of birth by cesarean section into PAXgene® Blood RNA tubes (Pre-Analytix, Hombrechtikon, Switzerland) which are designed for blood collection and storage of nucleic acids for later extraction and quantification. We determined the fetal hypoxic status in the preterm growth restriction cohort by collecting umbilical cord artery blood from the placenta at birth and measuring the pH, where hypoxia was defined as pH < 7.2 and normoxia being pH > 7.2. The pH was measured using clinical grade assays used in the maternity service at the different recruiting hospitals.

We also collected blood into Paxgene tubes from 42 controls where samples were collected at 28 and 34 weeks’ gestation. All these pregnancies had appropriately grown fetuses that progressed to birth at term. See Table 1 for participant characteristics in the FOX cohort.

**Table 1:** Patient characteristics for cases of preterm fetal growth restriction and control cohorts as part of the FOX study

Characteristics	Preterm fetal Growth restriction (*n* = 128)	Controls (*n* = 42)	*P*
Maternal age, years	32 (6)	30 (6)	0.13
Nulliparity	80 (63%)	38 (45%)	0.013
Body-mass index, kg/m^2^	27 (6)	24 (5)	0.0009
Smoking during pregnancy	17 (13%)	6 (7%)	0.16
Diabetes during pregnancy	15 (12%)	8 (10%)	0.60
Chronic hypertension	12 (9%)	0 (0%)	0.004
Preeclampsia	63 (49%)	3 (4%)	< 0.00001
Absent or reversed end diastolic flow in umbilical artery	58 (45%)	–	–
Median gestational age at blood sampling, weeks	30.5 (28.6–32.1)	30.0 (28–32.1)	0.21
Median gestational age at delivery, weeks	30.5 (28.6–32.1)	39.4 (39–40.2)	< 0.00001
Birthweight, g	1023 (315)	3594 (480)	< 0.00001
Median birthweight centiles, corrected for gestation^a^	0.1 (0.0–0.4)%	40.8 (27.9–60.9)%	< 0.00001
Male sex	71 (55%)	46 (55%)	0.92
Median Umbilical pH	7.27 (7.22–7.3)	–	
Umbilical pH < 7.2	22 (17.2%)	–	
Composite of severe neonatal morbidity^b^	39 (30%)	0 (−)	0.0002
Neonatal death within 42 days of birth	2 (2%)	0 (−)	0.42
Required resuscitation at birth^c^	106 (84%)	4 (9.5%)	< 0.0001
Median duration of stay in the neonatal intensive care unit (in days)	30 (10.8–61.5)	0 (0–0)	< 0.0001
Respiratory distress syndrome	94 (73%)	2 (5%)	< 0.0001
Required ventilation	86 (67%)	0 (−)	< 0.0001

Data are *n* (%), mean (SD), or median (interquartile range). Comparison between preterm fetal growth restriction cases and controls (where bloods were taken around the same gestational age) is by chi-squared analysis and *t* test. Each control contributed two blood samples for the analysis, at 28 and 32 weeks’ gestation. This was done to correct for possible changes in RNA concentrations across gestational age

^a^We used a fetal weight reference charts to determine centiles (Hadlock formula, except fetal sex was corrected for)

^b^Composite of severe neonatal morbidity includes intraventricular hemorrhage, periventricular leukomalacia, sepsis, necrotizing enterocolitis, seizures, or hypoxic ischemic encephalopathy

^c^Required resuscitation at birth which included any of the following: cardiopulmonary resuscitation, intubation, continuous positive airway pressure, and the administration of adrenaline or surfactant

#### Cohort of pregnant women who had paired blood samples taken pre and post steroids

We recruited 16 participants presenting to Mercy Hospital for Women and were administered 11.4-mg intramuscular dose of betamethasone. This injection is offered as part of clinical management for pregnancies where there is a risk of preterm birth occurring as it can improve perinatal outcomes for fetuses who deliver early. We obtained blood prior to the corticosteroid injection and 24 h to examine whether betamethasone affected the expression of circulating mRNAs. We obtained written informed consent from participants. Ethics approval was obtained for this study (R11/34).

#### EVERREST cohort

Maternal blood samples were collected from 46 women carrying a severely growth-restricted preterm fetuses (20 + 0 to 20 + 6 weeks’ gestation) from four sites in Europe: 37 from the UK (University College London), four from Germany (University Medical Centre, Hamburg-Eppendorf), One from Spain (Maternal-Fetal Unit Hospital Clinic, Barcelona), and four from Sweden (Lund University Hospital and University Hospital Malmö). Blood was collected in Paxgene tubes for later analysis. The protocol for the EVERREST cohort has been published and provides detailed methods regarding the study [19]. The cases selected for this study were from the biobank component of this prospective study.

Controls were either women undergoing termination of pregnancy at preterm gestations for medical reasons (*n* = 9), or healthy ongoing pregnancies where bloods were taken preterm and they progressed to term gestation (*n* = 18). See Additional file 1: Table S1 for patient characteristics.

#### Stillbirth study

Maternal blood samples were from six women carrying a growth-restricted preterm fetus that ended in stillbirth at either the Mercy Hospital for Women or the Royal Women’s Hospital. For all cases, the fetuses were extremely small (or growth restricted) at a very preterm gestation where the prognosis for the fetus was poor. For all cases, a clinical decision was made (in consultation with the mother) for conservative management rather than to deliver the pregnancy. Serial blood samples were drawn until fetal demise was diagnosed. The presence of ongoing fetal cardiac activity was confirmed at the time of each blood sampling. Gestation matched healthy controls were obtained at both hospitals from pregnancies which resulted in a healthy liveborn neonate. Bloods for both cohorts were collected into PAX gene tubes for later RNA extraction and quantification. See Additional file 1: Table S2 for clinical information regarding this cohort.

### Blood sample collection and storage

Maternal peripheral whole blood (2.5 ml) were collected into PAXgene® Blood RNA tubes according to the manufacturer’s instructions. After collection, tubes were gently inverted (~ 8 times), stored upright at room temperature for between 24 and 72 h, frozen at − 20 °C for 24–72 h and subsequently transferred to storage at − 80 °C until extraction processing.

### RNA extraction and preparation

Total RNA was extracted in batches at either at the Mercy Hospital for Women (FOX study, corticosteroid and stillbirth cohorts) or the University College London (EVERREST study), using the PAXgene® Blood miRNA Kit (Pre-Analytix, Hombrechtikon, Switzerland) according to the manufacturer’s instructions. In brief, samples were thawed and centrifuged and pellets washed and re-suspended in buffer. Proteins were digested using proteinase K, and cellular debris removed. The supernatant of the resulting flow-through was collected and isopropanol added. Samples were then centrifuged through PAXgene RNA spin columns, and genomic DNA contamination was removed using DNAse digestion. Purified RNA was eluted, and the concentration of RNA was determined by NanoDrop® ND-8000 8-sample spectrophotometer (ThermoFischer Scientific, Waltham, USA).

### Next-generation sequencing to identify mRNAs in the maternal circulation

#### RNA-seq sample preparation and sequencing

RNA was quantified using the NanoDrop 1000 Spectrophotometer (Thermo Scientific) and RNA integrity assessed with the Agilent Bioanalyzer 2100 (Agilent Technologies). Illumina’s TruSeq stranded RNA sample preparation kit with globin depletion was used to prepare libraries for sequencing, performed by the Australian Genome Research Facility (Parkville, Australia). Libraries were pooled and clustered using the Illumina cBot system, followed by sequencing on the Illumina HiSeq 2000 platform to obtain 50 base pair single-end reads.

#### Quality control and data pre-processing

The FastQC software [https://www.bioinformatics.babraham.ac.uk/projects/fastqc/] was used to assess the quality of the raw sequence data. Sequences were then mapped to the human reference genome (hg19, from the GATK Resource Bundle https://gatk.broadinstitute.org/hc/en-us/articles/360035890811-Resource-bundle) using the Rsubread program [20], and gene-level counts were obtained by the featureCounts procedure [21]. Further analysis was undertaken using the edgeR [22] and limma [23] R/Bioconductor packages. Counts per million (CPM) were calculated for each gene to standardize for differences in library-size, and filtering was carried out to retain genes with a baseline expression level of at least 2 CPM in 2 or more samples. TMM normalization was applied [24]. Multidimensional scaling (MDS) plots based on the log2(CPM) were generated to show relationships between samples based on case status (cases of preterm fetal growth restriction or controls), the hospital where samples were collected (to examine the possibility of batch variability), and fetal sex and to check for the presence of outliers.

#### Differential expression analysis

Voom was used to transform the count data to log2CPM, estimate the mean-variance relationship, and compute appropriate observation-level weights. A linear model [25] was then fitted to this data, including a coefficient for the case vs controls difference. Moderated *t*-statistics were used to assess differential expression between case and control samples, with genes ranked according to their false discovery rate [26].

#### Bioinformatic analysis of sequencing data

Linear regression analysis identified 21 genes (or pseudogenes) that were differentially expressed, after adjusting for multiple comparisons (Table 2). The switchBox package from Bioconductor was used to train and validate a K-Top-scoring Pair classifier (KTSP). The KTSP algorithm is a simple binary classifier based on the ordering of two measurements, where the predictions are based on the gene expression data [27]. A classifier was trained using the default filtering function based on the Wilcoxon test, and the top scoring pairs were extracted. This switchBox analysis identified a further two genes that were differentially expressed (*RCBTB2* and *SKIL*).

**Table 2:** Circulating mRNAs that were differentially expressed in the maternal blood of prospective FOX cohort preterm fetal growth restriction vs healthy pregnancies identified by RNA-seq and linear regression analysis

Entrez ID	Gene symbol	logFC	Average expression	P	Adjusted P*	Selected for RT-PCR validation**
100462977	MTRNR2L1	− 2.26	3.71	4.55E−16	1.95E−12	
8460	TPST1	1.39	4.43	3.90E−19	4.38E−15	Y
9509	ADAMTS2	2.99	0.36	6.09E−11	2.36E−08	Y
100505479	NA	− 1.98	1.34	2.25E−12	2.02E−09	
23500	DAAM2	2.35	2.11	2.21E−11	1.13E−08	Y
144535	CFAP54	1.33	1.67	5.21E−16	1.9E−12	
4549	RNR1	− 1.89	8.60	1.34E−11	7.66E−09	
4550	RNR2	− 1.60	11.06	3.50E−11	1.48E−08	
100463486	MTRNR2L8	− 1.51	5.15	1.26E−11	7.42E−09	Y
100462981	MTRNR2L2	− 1.26	2.47	1.17E−13	1.88E−10	
100505635	LOC100505635	2.16	− 0.12	3.77E−09	7.28E−07	
4929	NR4A2	1.37	0.24	5.65E−12	3.96E−09	Y
10720	UGT2B11	− 1.37	0.53	1.09E−11	6.78E−09	Y
1831	TSC22D3	0.86	8.68	3.21E−22	7.21E−18	Y
2012	EMP1	1.24	0.65	2.51E−12	2.09E−09	Y
246	ALOX15	− 1.38	1.20	1.03E−10	3.71E−08	Y
100463488	MTRNR2L10	− 1.47	− 0.34	9.47E−10	2.35E−07	Y
55301	OLAH	2.07	0.41	8.43E−08	9.38E−06	Y
11251	PTGDR2	− 0.99	2.17	2.39E−14	4.87E−11	Y
25790	CFAP45	0.99	2.82	3.48E−14	6.51E−11	
5239	PGM5	1.24	1.67	1.62E−10	5.44E−08	Y

*Entrez ID* Entrez gene identifier, *logFC* log2 fold-change, *AveExpr* average expression, or the *A* value, *Y* Yes. *Adjusted *P* value corrected for the false discovery rate. **For RT-PCR validation, we selected these 13 genes because Taqman primer assays were available. The remaining eight genes we did not measure did not have Taqman primers available

### Validation with quantitative real-time reverse-transcription polymerase chain reaction (qPCR)

Total RNA was converted to cDNA using Applied Biosystems high capacity cDNA reverse transcriptase kit (Thermofisher). Quantitative real-time reverse-transcription polymerase chain reaction (qPCR) validation was carried out for 15 of the 23 genes identified in the NGS analysis (genes assessed were *RCBTB2* and *SKIL* which were identified from the switchBox analysis, and 13 out of 21 genes from the regression analysis and these are listed on Table 2) using specific FAM-labeled Taqman primer assays and universal PCR master mix (Thermofisher) on the CFX 384 (Bio-Rad, Hercules, CA) with the following run conditions: 50 °C for 2 min, 95 °C for 10 min, 95 °C for 15 s, and 60 °C for 1 min (40 cycles). *GUSB, YWHAZ*, and *B2M* were used as reference housekeeping genes after validating that their expressions were not altered across samples (collection timing, location, and extraction). The comparative ΔΔCT method of analysis was used to determine relative expression. A CT value over 35 cycles was used as a threshold to deem the transcript as undetectable.

### Statistical analysis of qPCR data and clinical baseline characteristics

To compare baseline clinical characteristics of continuous data, either Student’s *T* test (normally distributed) or the Mann-Whitney test (not normally distributed) was used. Chi-squared test was used to compare categorical data.

For the qPCR results, data was tested for normal distribution and statistically analyzed as appropriate. When three or more groups were compared, the 1-way ANOVA (for parametric data) or Kruskal-Wallis test (for non-parametric data) was used. When two groups were compared, either Student’s *T* test or the Mann-Whitney test was used. All qPCR data is expressed as mean ± SEM. *P* < 0.05 was considered significant.

To combine data from RNAs to develop potential multi-marker tests, multivariate logistic modeling was performed and discrimination assessed using area under ROC curves and the sensitivity generated by setting specificity at around 90% (where it equates to a 10% screen positive rate).

Statistical software used was Stata v15 (StataCorp. 2017. *Stata Statistical Software: Release 15*. College Station, TX: StataCorp LLC) or Graphpad Prism 7 (GraphPad Software, LA Jolla, CA).

## Results

### FOX study: prospective study of pregnancies complicated by preterm fetal growth restriction

We performed a multi-center prospective cohort study of 128 participants with pregnancies complicated by preterm fetal growth restriction across six major tertiary referral hospital across Australia and New Zealand (Fetal Oxygenation (FOX) study). Table 1 provides the baseline clinical characteristics of study participants in the FOX study and shows that the preterm fetal growth restriction cohort had significantly poorer neonatal outcomes compared to controls.

All participants had an ultrasound examination where the estimated fetal weight (EFW) was < 10th centile corrected for gestation. They all subsequently gave birth by cesarean section < 34 weeks’ gestation between 25 (+ 2 days) and 34 (+ 0 days) weeks where the decision for iatrogenic delivery was triggered by concerns regarding fetal wellbeing. For all these cases of preterm fetal growth restriction, we obtained maternal whole blood within 2 h prior to birth. In fact, most of the cases were very significantly growth restricted with a median birthweight centile (using intrauterine fetal charts [28]) of only 0.1 (interquartile range 0.0–0.4), well below the 10th centile.

Blood samples were also collected from 42 controls which were normal pregnancies where samples were taken at similar gestations as those obtained from the preterm fetal growth-restricted cohort. Given they were healthy pregnancies, the control cohort were not delivered around the time of blood collection but, instead, progressed uneventfully to term delivery (> 37 weeks’ gestation).

### RNA-seq to identify circulating RNA transcripts that are differentially regulated with severe preterm fetal growth restriction

**Fig. 1:**
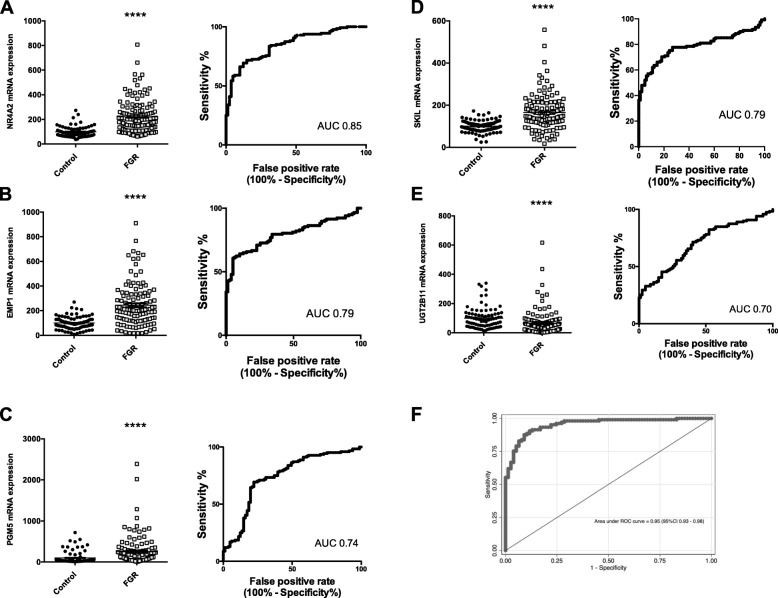
Expression of circulating mRNAs among pregnancies with preterm fetal growth restriction in the FOX cohort. **a–e** qRT-PCR expression of five genes identified by RNA-seq, and the receiver operating characteristic curves. Controls were ongoing pregnancies unaffected by growth restriction where bloods were collected around the same gestational ages. **f** The receiver operating characteristic curve for detecting preterm fetal growth restriction when these circulating genes were combined as a potential diagnostic test. FGR, fetal growth restriction. ****p < 0.0001. Error bars are mean ± SEM

We performed RNA-seq on RNA isolated from whole blood from the FOX cohort study participants. Bioinformatic linear regression analysis identified 21 genes or pseudogenes that were differentially expressed between those with preterm fetal growth restriction and controls (Table 2). We also performed a switchBox analysis which identified a further two genes, *RCBTB2* and *SKIL*. We performed a technical validation of 15 of the 23 genes identified by the RNA-seq analysis using quantitative RT-PCR (13 selected from Table 2 and the two identified by the switchBox analysis) and confirmed dramatic differences in expression for 13 of these genes (Fig. 1a–e and Additional file 1: Fig. S1). Of two genes identified as differentially expressed in the RNA-seq analysis that we selected for PCR validation, MTRNR2L8 was not significantly different and MTRNR2L10 had either low or undetectable levels of expression in most samples (data not shown).

Corticosteroids are routinely administered to the mother prior to a planned birth before 34 weeks as this substantially improves perinatal survival and neonatal outcomes [29]. Given all those (except one) in the preterm fetal growth restriction cohort were exposed to antenatal corticosteroids but none of the controls were, we were concerned that the exposure to corticosteroids might explain the differences seen in circulating RNA. Therefore, we collected paired blood just prior to, and 24 h after a corticosteroid injection from 16 pregnant women. This group was separate to the FOX study. Of the 13 genes that were differentially expressed in the blood of women with preterm growth restriction by qRT-PCR, the five shown in Fig. 1 (*NR4A2*, *EMP1*, *PGM5*, *SKIL*, and *UGT2B1*) were not affected by corticosteroid administration (Additional file 1: Fig. S2), while the eight genes shown in Fig. S1 were changed by the administration of corticosteroids (the effects of corticosteroids on these eight genes are shown in Additional file 1: Fig. S3).

Using the qRT-PCR findings for the five genes not affected by corticosteroid administration, we performed multivariable logistic regression to develop a multi-marker screening test for preterm fetal growth restriction. This yielded a potential clinical test with an area under the receiver operating characteristic (ROC) curve of 0.95 (Fig. 1f). At a specificity of 90.9% (i.e., a 10% screen positive rate), the test had 87.6% sensitivity in identifying preterm fetal growth restriction, with a positive likelihood ratio of 9.6. When 10-fold cross validation was applied to correct for overfitting, the ROC was 0.94 and the sensitivity was 72% at a similar specificity of 90.2%.

Forty-nine percent of the cases of preterm fetal growth restriction had co-existent preeclampsia in the FOX cohort (Table 1). To confirm the fact that these five genes remained differentially expressed in the absence of preeclampsia, we split the cases into two groups; those with and without co-existent preeclampsia and compared them to controls. We confirmed that all five genes remained differentially expressed in pregnancies with preterm fetal growth restriction whether or not there was co-existent preeclampsia (Additional file 1: Fig. S4).

Hence, by applying RNA-seq on a large cohort, we have identified 13 genes that are differentially expressed in the circulation in pregnancies complicated by preterm fetal growth restriction. Among these, circulating RNA transcripts of five genes were not affected by maternal corticosteroid administration: *NR4A2*, *EMP1*, *PGM5*, *SKIL*, and *UGT2B1*. Combining these biomarkers generated potential tests with strong diagnostic performance to detect the presence of early preterm fetal growth restriction, although they would need to be validated in further studies.

### Validating the association between circulating RNA coding *NR4A2*, *EMP1*, *PGM5*, *SKIL*, and *UGT2B1* and preterm fetal growth restriction

We next set out to validate the association between the five genes unaffected by maternal corticosteroid administration and preterm fetal growth restriction in the EVERREST cohort from Europe [19]. The samples investigated were the first samples collected from 46 participants soon after the initial diagnosis of preterm fetal growth restriction (EFW < 3rd centile, median gestational age of sample collection was 23 + 1 weeks of gestation (interquartile range (IQR) 22 + 2 to 24 + 4 weeks’ gestation)). Controls were fetuses that were appropriately grown for gestational age (Additional file 1: Table S1 shows the clinical characteristics of the EVERREST cohort). All samples in the EVERREST cohort were collected prior to the administration of antenatal corticosteroids and were therefore not exposed to this drug, and neither were any of the controls.

**Fig. 2:**
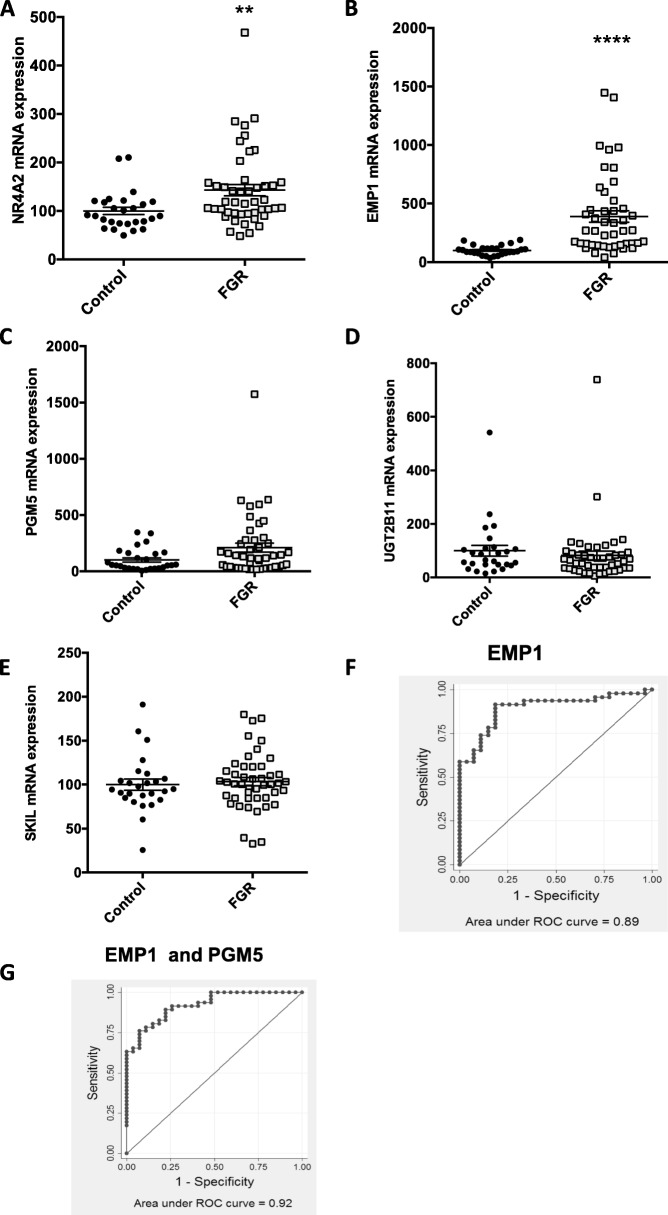
Expression of circulating mRNAs among 46 pregnancies with preterm fetal growth restriction in the EVERREST cohort vs 27 appropriately grown fetuses. **a–e** Expression levels of five circulating RNAs that were discovered in the FOX cohort. For *PGM5 p* = 0.06. **f** The receiver operating characteristic curve for *EMP1 t*o detect fetal growth restriction. **g** The receiver operating characteristic curve when *EMP1* and *PMG5* are combined to detect fetal growth restriction. **p < 0.004. ****p < 0.0001. Error bars are mean ± SEM

RT-PCR analysis confirmed that the mRNA expression of *NR4A2* and *EMP1* were significantly increased with preterm fetal growth restriction (Fig. 2a and b) and *PGM5* expression trended towards an increase (*p* = 0.06, Fig. 2c). *UGT2B1* (Fig. 2d) and *SKIL* was not differentially expressed (Fig. 2e).

The association between *EMP1* and preterm fetal growth restriction appeared strongest. In this validation cohort, circulating *EMP1* as a lone marker had 65% sensitivity in identifying the presence of preterm fetal growth restriction at 90% specificity, and an AUC of 0.89 (95% CI 0.82–0.97; Fig. 2f). Combining *EMP1* and *PGM5* had 72% sensitivity to detect preterm fetal growth restriction at 90% specificity, and an AUC of 0.92 (95% CI 0.86–0.98, Fig. 2g).

Thus, we have validated the association between preterm fetal growth restriction and increased circulating mRNA coding *NR4A2* and *EMP1* (and possibly *PGM5*) in an independent cohort of samples from another continent.

### Identifying circulating transcripts that are associated with in utero fetal acidemia among fetuses that are growth restricted in the FOX cohort

A clinical test that can identify preterm growth-restricted fetuses at high risk of acidemia in utero and requiring urgent delivery could be useful to monitor fetal wellbeing. We examined the association between mRNA concentrations of the 13 genes we identified as differentially expressed in the blood of women with preterm growth-restricted pregnancies (shown in Fig. 1 and Additional file 1: Fig. S1) with the umbilical artery pH levels at the time of birth in the FOX cohort.

For all of the cases of preterm fetal growth restriction in the FOX cohort, the maternal blood was collected within 2 h prior to birth, and the umbilical artery pH was sampled shortly after birth. The umbilical artery blood was obtained from the umbilical cord (attached to the delivered placenta) at birth. The umbilical artery pH reflects the acidemic status of the fetus during its last moments prior to birth and is widely used clinically as a gold standard to retrospectively determine the true degree of fetal acidemia in utero [30]. We did not restrict this analysis to the five genes unaffected by corticosteroids given the entire cohort in this analysis (except one) was exposed to this medication.

**Fig. 3:**
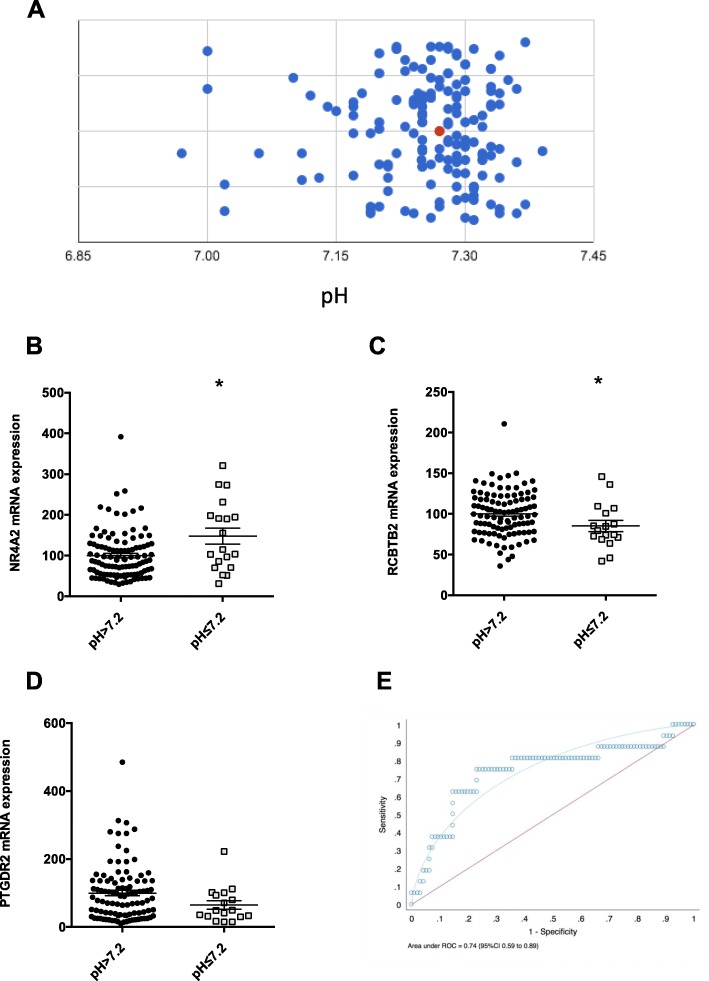
The association between circulating mRNAs in the maternal circulation and fetal acidemia. Those with preterm fetal growth restriction in the FOX study were examined. Maternal bloods were taken within 2 h of birth and fetal pH (pH < 7.2 is defined as fetal acidemia) taken from the umbilical artery shortly after birth. **a** The spread of umbilical artery pH concentrations in the FOX cohort. Red dot is the median value. **b–d** Expression levels of three circulating RNAs. For *PTGDR2 p* = 0.07. **e** The receiver operated curve when circulating *NRA2*, *RCBTB2*, and *PTGDR2* in the maternal circulation are combined to detect fetal acidemia (pH < 7.2). The area under the receiver operated curve (AUC) was 0.74 (95% CI 0.59–0.89). *p < 0.05

We defined fetal acidemia as an umbilical artery blood pH < 7.2 given this is associated with a 4.2-fold increased risk of perinatal death (compared to those born with a pH > 7.2) [9] and is therefore a clinically significant threshold. Of the 128 cases of preterm growth restriction, 22 (17.2%) had a pH < 7.2. Figure 3a shows the spread of umbilical cord pH among the preterm fetal growth restriction cohort.

Of the 13 genes, circulating mRNA coding *NR4A2* (Fig. 3b) and *RCBTB2* (Fig. 3c) were differentially expressed among growth-restricted fetuses that were acidemic at birth. *PTGDR3* (Fig. 3d) trended towards a reduction (*p* = 0.07). Combining the mRNA expression of these three genes generated a multi-marker test with an AUC of 0.74. Thus, we have identified a panel of circulating mRNA transcripts that are associated with the presence of significant fetal acidemia in utero.

### Correlating circulating mRNA transcripts and the degree of placental insufficiency in the EVERREST cohort

To obtain further evidence that circulating mRNAs may be associated with clinical parameters that reflect fetal wellbeing, we correlated the circulating mRNAs with pathological Doppler ultrasound features of placental insufficiency: increased blood flow resistance in the uterine and umbilical arteries, and decreased resistance in the fetal middle cerebral arteries. We performed this study using the EVERREST cohort where the samples were collected soon after the diagnosis of preterm fetal growth restriction and prior to steroid administration. We measured three of the most promising candidates by PCR: *NR4A2*, *RCBTB2* since they were differentially expressed with fetal acidemia in the FOX cohort, and *EMP1* given its particularly strong association with fetal growth restriction in both the FOX (Fig. 1b) and EVERREST cohorts (Fig. 2b, f).

**Fig. 4:**
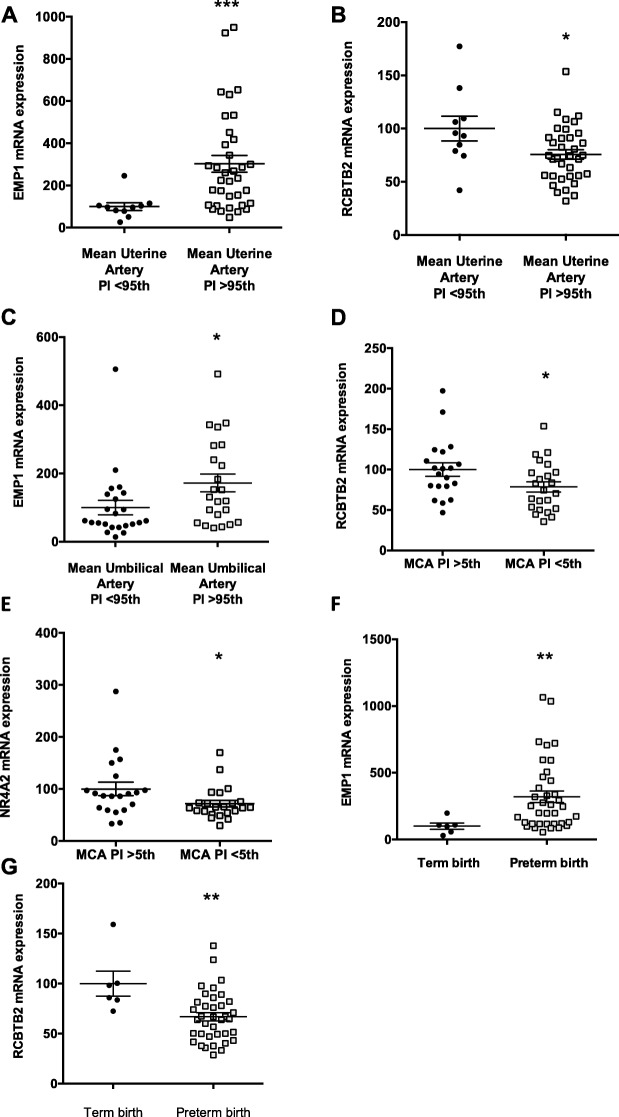
The association between circulating mRNAs in the maternal circulation and clinical parameters that reflect placental insufficiency. Those with preterm fetal growth restriction in the EVERREST study were examined. Circulating levels of **a***EMP1* and **b***RCBTB2* and uterine artery blood flow Pulsatility Index (PI), where >95th centile is a threshold clinical to suggest increased blood flow resistance that occurs with increased placental insufficiency. **c** Circulating levels of *EMP1* and the umbilical artery (UA), where > 95th PI is abnormally increased (also reflects placental insufficiency). **d** Circulating levels of *RCBTB2* and **e***NR4A2* and the middle cerebral artery (MCA), where < 5th PI is abnormal and suggests fetal circulatory redistribution that occurs when fetuses are coping with placental insufficiency. **f** Circulating levels of *EMP1* and **g***RCBTB2* at initial recruitment grouped to whether the pregnancy was delivered preterm or progressed to term gestation (> 37 weeks’ gestation). *p < 0.05, **p < 0.01, ***p < 0.001. Error bars are mean ± SEM

Of the three, circulating *EMP1* (Fig. 4a) and *RCBTB2* (Fig. 4b) was significantly changed with raised uterine artery blood flow resistance. *EMP1* (Fig. 4c) was associated with increased umbilical artery Doppler blood flow resistance, but *RCBTB2* and *NR4A2* were not (not shown). However, *RCBTB2* (Fig. 4d) and *NR4A2* (Fig. 4e) were associated with decreased middle cerebral artery resistance.

Finally, we examined the prognostic ability of the circulating transcripts from the first blood sample obtained soon after diagnosis (taken between 20 + 0 to 26 + 0 weeks’ gestation) to predict which pregnancies will progress to term gestation (which implies the presence of a less severe form of placental insufficiency at the time of blood sampling that is also not progressive) versus those that required preterm delivery due to concerns of deteriorating fetal status. We did this by examining those recruited to the EVERREST study and grouping the cohort according to whether they progressed to term gestation (> 37 weeks’ gestation) or not. *EMP1* (Fig. 4f) was significantly increased among those destined for preterm birth, and *RCBTB2* was significantly decreased (Fig. 4g).

Thus, we have shown circulating mRNAs that are associated with clinically accepted parameters of placental insufficiency. *EMP1* appears to consistently demonstrate the strongest association.

### Identifying circulating transcripts that are associated with subsequent stillbirth

We next measured the 13 gene transcripts in blood that we identified as differentially expressed in association with preterm fetal growth restriction among six cases that ended in stillbirth (Additional file 1: Table S2 shows the clinical characteristics). These were pregnancies where it was clinically apparent that growth restriction was exceptionally severe. In these cases, the prognosis was deemed exceptionally poor because the fetus was both extremely growth restricted and preterm, and thus on the threshold of viability. In all cases, a clinical decision was made with the parents not to deliver the fetuses but instead proceed with expectant management in the hope of gaining a meaningful increase in gestation. None received antenatal corticosteroids. All fetuses were confirmed to be live at the time of sample collection, evidenced by the presence of fetal cardiac activity. Controls were healthy, ongoing pregnancies.

**Fig. 5:**
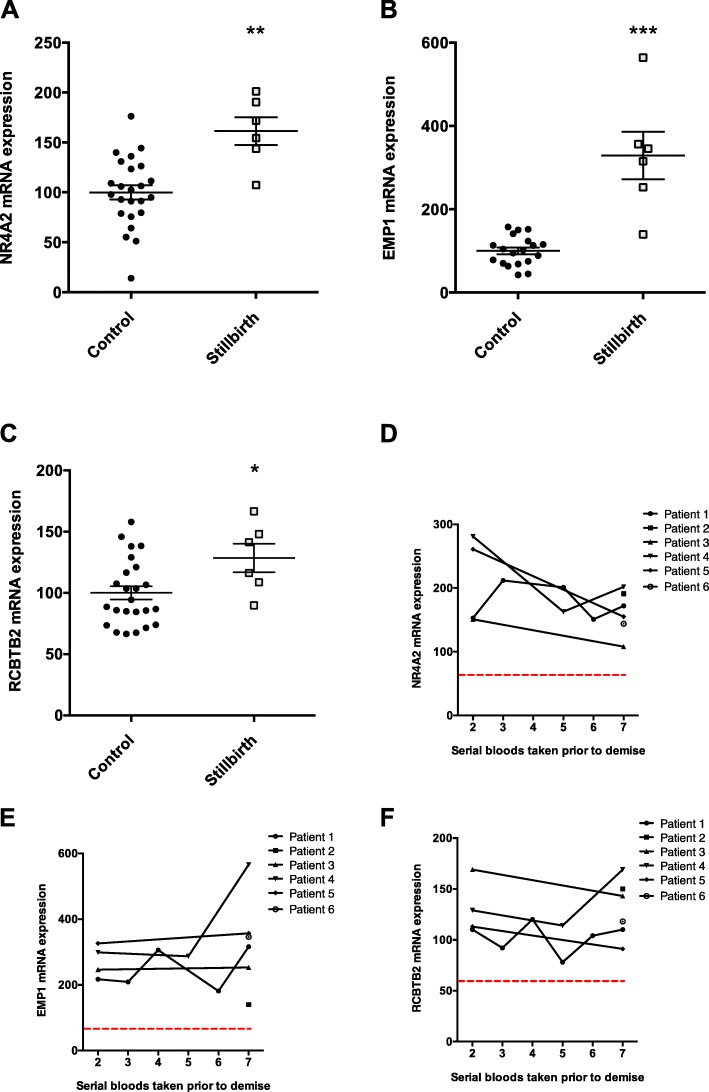
The association between circulating mRNAs in maternal circulation and future stillbirth. **a–c** Expression levels of three circulating mRNAs among women with a live pregnancy at the time of blood sampling but later had a stillbirth. These were the last bloods taken closest to demise. **d–f** Serial blood samples taken from these same cases of stillbirth prior to fetal demise (one sample taken for two patients; two samples taken across 5 and 14 days for two patients; three samples taken across 10 days for one patient, and 6 bloods taken across 36 days for one patient). Red dotted line represents the median mRNA expression levels of the control group (the same controls as shown in **a**–**c**). These controls were healthy ongoing pregnancies where bloods were taken at similar gestations. *p < 0.05, **p < 0.01, ***p < 0.001. Error bars are mean ± SEM

We first examined the final bloods taken prior to fetal demise. Of the 13 genes, circulating mRNA coding *EMP1* (Fig. 5a), *NR4A2* (Fig. 5b) and *RCBTB2* (Fig. 5c) were significantly altered in the stillbirth cohort compared to controls. The differences appeared particularly strong for *EMP1* where five of six cases had mRNA expression levels higher than all controls.

We then examined the expression of these three genes in all serial blood samples taken prior to demise in the same population and found that expression levels seemed to be consistently higher than the median levels in the control group (Fig. 5d–f).

## Discussion

By performing RNA-seq on samples from a large multi-center cohort study, we have identified numerous circulating mRNA that were very significantly deranged in the circulation of pregnancies complicated by early preterm fetal growth restriction. Importantly, we have also obtained evidence that the relative concentrations of mRNAs of some genes, notably *EMP1*, correlated with many clinical parameters that reflect severity of placental dysfunction and the likely presence of in utero fetal acidemia [9]. These include umbilical cord artery pH at birth, and Doppler waveform velocimetry of the uterine, umbilical artery, and middle cerebral arteries. Furthermore, we have also identified circulating mRNAs that were deranged in a small cohort of pregnancies destined for stillbirth. Together, our work suggests that circulating mRNAs could be used to monitor fetal health dynamically over time and to identify fetuses with very severe placental insufficiency and at high risk of imminent stillbirth. It is possible that a circulating mRNA-based test could be developed that allows clinicians to deliver severely compromised fetuses before demise, maximizing gestation yet preventing cases of stillbirth.

Of all the circulating mRNA molecules we identified, *EMP1* appears the most promising as a marker of placental insufficiency. Even as a lone marker, it seems to have strong discriminatory ability to identify the presence of early preterm fetal growth restriction, a finding that we validated in the EVERREST cohort from Europe [19]. Furthermore, *EMP1* was correlated with severe clinical parameters that reflect placental insufficiency and was deranged in another cohort of women with preterm fetal growth restriction whose pregnancies ended in stillbirth. Circulating *EMP1* (and perhaps *RCBTB2)* merit further study to determine whether they can add to the diagnostic accuracy of ultrasound to monitor fetal health.

There is little known about the five genes we identified as differentially regulated in association with placental insufficiency (Additional file 1: Table S3 summarizes the postulated biological functions of the protein products coded by *NR4A2*, *EMP1*, *PGM5*, *SKIL*, and *UGT2B1* mRNAs).

While the concept of measuring circulating molecules (including mRNA) to predict pregnancy complications has been around for over a decade [16], the idea of measuring mRNA to monitor fetal health and placental insufficiency to prevent stillbirth is novel. Indeed, none of the circulating mRNA markers we report here have been previously reported to be associated with placental insufficiency.

In 2016, a consortium published the Delphi definition for fetal growth restriction [31]. The reason why we did not use this definition for the FOX cohort is that the design of the FOX study predated this publication. However, for our growth restriction cohort, the birthweight centiles of cases were mostly below < 3rd centile and had ultrasound findings that triggered birth. On reviewing the clinical information on the cohort, 97% (124 out of the 128 cases) fulfilled the Delphi criteria for fetal growth restriction. All the cases of preterm growth restriction in the EVERREST study fulfilled the Delphi definition for fetal growth restriction.

Our work has a number of strengths. Preterm fetal growth restriction represents one of the most dangerous complications of pregnancy for the fetus but is uncommon. Given its rarity, we had to recruit from multiple referral centers to obtain sufficient numbers. Furthermore, the protocol for the FOX study where we collected blood within 2 h of birth so we could contemporaneously correlate circulating mRNA findings with umbilical cord pH may be considered novel. An important further strength is that we validated findings in the FOX study in an independent preterm fetal growth restriction cohort from Europe.

One limitation is that we had anticipated we would obtain a larger number of fetuses that were acidemic at birth in the FOX cohort. The fact that only 22 of the cohort with preterm fetal growth restriction were significantly acidemic at birth (pH < 7.2) meant we were likely to be underpowered to find significant hits using the RNA-seq data. However, by examining the RT-PCR quantitative data on the 13 genes that were differentially expressed with preterm fetal growth restriction, we generated a multi-marker test with an AUC of 0.72. This suggests that it remains possible a circulating mRNA signature exists that can identify fetal acidemia, but further development of a clinical test would require larger studies so a cohort of fetuses born acidemic is recruited in sufficient numbers for adequately powered studies. While possible, this would be a challenging cohort to collect.

We have provided robust evidence in three cohorts that circulating mRNA signatures are altered where there is placental insufficiency, severe placental dysfunction including fetal hypoxia, and even impending stillbirth. We hope it will generate interest for new studies to further develop an mRNA signature that could help clinicians identify which fetuses are truly acidemic in utero and need urgent birth, compared to those who are not compromised and may be left safely in utero to gain gestation and advance fetal development. Such future studies would need to identify a test that combines the expression of different mRNAs that has strong performance characteristics for placental insufficiency, lock down an algorithm, and validate the test in a prospective validation study. Furthermore, these studies would also need to demonstrate strong associations with important clinical outcomes such as severe neonatal complications and optimally, also neurodevelopmental outcomes. If developed, such a test could decrease stillbirths and improve perinatal and long-term outcomes.

## Supplementary information

**Additional file 1 Supplementary Table 1.** Patient characteristics for the cases of Fetal Growth Restriction (FGR) and control cohorts as part of the EVVEREST study. **Supplementary Table 2.** Patient characteristics for the cohort of stillbirths and controls. **Supplementary Table 3.** Summary of the postulated biological functions of the proteins encoded by the five genes that were differentially regulated in the FOX cohort. **Supplementary Fig. S1.** Expression of circulating mRNAs among pregnancies with preterm fetal growth restriction in the FOX cohort. A-H shows qRT-PCR expression of eight genes identified by RNA-seq, and their respective receiver operating characteristic (AUC) curves. Controls were ongoing pregnancies unaffected by growth restriction where bloods were collected around the same gestational ages. **** *p* < 0.0001. Error bars are mean ± SEM. **Supplementary Fig. S2.** Expression of five circulating mRNAs among 16 women who had bloods taken just prior to (Pre), and 24 h after (Post) an intramuscular injection of corticosteroid (11.4 mg of betamethasone). These are the same five mRNAs as those shown in Fig. 1 of the main manuscript. None were significantly different after the injection of corticosteroids. Error bars are mean ± SEM. **Supplementary Fig. S3.** Expression of eight circulating mRNAs among 16 women who had bloods taken just prior to (Pre), and 24 h after (Post) an intramuscular injection of corticosteroid (11.4 mg of betamethasone). These are the same eight mRNAs as those shown in supplementary Fig. S1. ** *p* < 0.01, *** *p* < 0.001, **** *p* < 0.0001. Error bars are mean ± SEM. **Supplementary Fig. S4.** Expression of the five circulating mRNAs in the FOX cohort where the cases of preterm fetal growth restriction were split according to whether there was co-existent preeclampsia, or not.

## Data Availability

The RNA seq data has not been deposited on a freely downloaded repository given it has sequence information that could identify participants. The authors are happy to be contacted where the data could be made available if confidentiality agreements are signed between institutions.

## Abbreviations

mRNA: Message ribonucleic acid
FOX: Fetal Oxygenation Study
EFW: Estimated fetal weight
RT-PCR: Reverse transcriptase-polymerase chain reaction
qPCR: Quantitative polymerase chain reaction
ROC: Receiver operated curve
AUC: Area under the curve
*NR4A2*: Nuclear receptor-related 1 protein
*EMP1*: Epithelial membrane 1 protein
*PGM5*: Phosphoglucomutase-like protein 5
*SKIL*: Ski-like protein
*UGT2B1*: UDP-glucuronosyltransferase 2B1 precursor
*RCBTB2*: RCC1 and BTB domain-containing protein 2
*MT4NR2L10*: MT-RNR2-like 10
*MT4NR2L8*: MT-RNR2-like 8
