# Supplementary material for: Circulating mRNAs are differentially expressed in pregnancies with severe placental insufficiency and at high risk of stillbirth
Source: BMC Med. 2020 May 22;18:145. doi: 10.1186/s12916-020-01605-x (PMC7243334; doi:10.1186/s12916-020-01605-x)
Supplement: Supplementary file 1 — Additional file 1 Supplementary Table 1. Patient characteristics for the cases of Fetal Growth Restriction (FGR) and control cohorts as part of the EVVEREST study. Supplementary Table 2. Patient characteristics for the cohort of stillbirths and controls. Supplementary Table 3. Summary of the postulated biological functions of the proteins encoded by the five genes that were differentially regulated in the FOX cohort. Supplementary Fig. S1. Expression of circulating mRNAs among pregnancies with preterm fetal growth restriction in the FOX cohort. A-H shows qRT-PCR expression of eight genes identified by RNA-seq, and their respective receiver operating characteristic (AUC) curves. Controls were ongoing pregnancies unaffected by growth restriction where bloods were collected around the same gestational ages. **** p < 0.0001. Error bars are mean ± SEM. Supplementary Fig. S2. Expression of five circulating mRNAs among 16 women who had bloods taken just prior to (Pre), and 24 h after (Post) an intramuscular injection of corticosteroid (11.4 mg of betamethasone). These are the same five mRNAs as those shown in Fig. 1 of the main manuscript. None were significantly different after the injection of corticosteroids. Error bars are mean ± SEM. Supplementary Fig. S3. Expression of eight circulating mRNAs among 16 women who had bloods taken just prior to (Pre), and 24 h after (Post) an intramuscular injection of corticosteroid (11.4 mg of betamethasone). These are the same eight mRNAs as those shown in supplementary Fig. S1. ** p < 0.01, *** p < 0.001, **** p < 0.0001. Error bars are mean ± SEM. Supplementary Fig. S4. Expression of the five circulating mRNAs in the FOX cohort where the cases of preterm fetal growth restriction were split according to whether there was co-existent preeclampsia, or not. [file 12916_2020_1605_MOESM1_ESM.docx]

**Additional File 1: Supplementary Appendix**

**This appendix has been provided by the authors to give readers additional information about their work**

Supplement to: Hannan NJ et al., Circulating mRNAs are differentially expressed in pregnancies with severe placental insufficiency and at high risk of stillbirth.

**Supplementary Table 1: Patient characteristics for the cases of Fetal Growth Restriction (FGR) and control cohorts as part of the EVVEREST study.**

|  | **Preterm fetal growth restriction**  **(n=46)** | **Controls (n= 27)** | ***P*** |
| --- | --- | --- | --- |
| Maternal age, in years | 32 (5.5) | 32.6 (5.8) | 0.76 |
| Nulliparity n (%) | 29 (63%) | 11/17 (64%)* | 0.90 |
| Body-mass index, kg/m^2^ | 26 (5) | 26 (7) | 0.36 |
| Smoking during pregnancy | 4 (9%) | 3 (11%) | 0.69 |
| Diabetes | 4 (9%) | 2 (7%) | 1.0 |
| Chronic hypertension | 5 (11%) | 0 | 0.16 |
| Preeclampsia | 5 (11%) | 0 | 0.16 |
| Absent or reversed end diastolic flow in umbilical artery | 12 (26%) | - | - |
| Gestational age at sample collection in weeks - median (IQR) | 23.4 (23 – 24) | 25.8 (24.5 – 27.1) | 0.001 |
| Gestational age at birth in weeks - median (IQR) | 30 (28.4 – 31.3) | 34 (30.7 – 37.2) | 0.028 |
| Male sex | 16 (35%) | - | - |
| Birthweight (g) median (IQR). | 864**  (510-1842) | 3350  (3170 – 3745) | <0.0001 |

Data are n (%), mean (SD), or median and Interquartile range (IQR). Comparisons between FGR cases and gestation matched controls were done using either the χ2 analysis or *t*-test. * Information on parity for nine controls were not available. ** Birthweight was restricted to the 34 cases of preterm fetal growth restriction that were livebirths.

**Supplementary Table 2: Patient characteristics for the cohort of stillbirths and controls**

|  | **Stillbirth**  **(n=6)** | **Controls**  **(n=25)** | ***P*** |
| --- | --- | --- | --- |
| Maternal age, in years | 25.5 (19.5-36.0) | 30 (27.5-33.0) | 0.76 |
| Nulliparity – n (%) | 4 (67%) | 16 (64%) | 0.83 |
| Smoking during pregnancy | 1 (20%)* | 1 (4%) | 0.31 |
| Diabetes | 0 | 2 (4%) | - |
| Chronic hypertension | 1 (17%) | 1 (4%) | 0.36 |
| Preeclampsia | 1 (17%) | 0 | - |
| Absent or reversed end diastolic flow in umbilical artery | 5 (83%) | 0 | - |
| Gestational age at sample, weeks (IQR) | 27.4 (23.1-28.1) | 26.3 (24-29.5) | 0.75 |
| Gestational age at delivery, weeks | 27.7 (25.7-29.5) | 39.4 (38.7-40.6) | <0.0001 |
| Birthweight – grams | 452 (310-696.3) | 3468 (3190-3710) | <0.0001 |

Data are n (%), or median (IQR). Comparison between Stillbirth cases and controls is by non-parametric Mann-Whitney-U test due to small n number. *Information for one case was missing (hence 20% is 1 out of 5).

**Supplementary Table 3: Summary of the postulated biological functions of the proteins encoded by the five genes that were differentially regulated in the FOX cohort.**

| **Gene** | **Protein name of the gene product** | **Postulated biological function** |
| --- | --- | --- |
| ***NR4A2*** | Nuclear Receptor Subfamily 4 Group A Member 2 | Also known as NURR1, it is involved with regulation of inflammation; overexpression leads to a decrease in pro-inflammatory cytokines and chemokines. |
| ***EMP1*** | Epithelial membrane protein -1 | Also known as Tumour-Associated Membrane Protein. It is primarily expressed in squamous epithelium and plays roles in tumour cell survival, although this varies depending on tumour type. A histological marker of poor prognosis in paediatric leukemias. |
| ***PGM5*** | Phosphoglutamase 5 | Enzyme involved in carbohydrate metabolism. Also involved in the structural regulation of adherens-type cellular junctions which interact with the cytoskeleton. |
| ***SKIL*** | Ski-like protein | Also known as *SnoN*, it has been reported to act as a proto-oncogene by negatively regulating TGF-β. SKIL regulates mammary gland alveologenesis and onset of lactation by promoting prolactin/Stat5. |
| ***UGT2B11*** | UDP glucuronosyltransferase 2 family | There is very little published on this protein. It may play a role in steroidogenesis. |

**Supplementary Figure S1:** Expression of circulating mRNAs among pregnancies with preterm fetal growth restriction in the FOX cohort. **A-H** shows qRT-PCR expression of eight genes identified by RNA-seq, and their respective receiver operating characteristic (AUC) curves. Controls were ongoing pregnancies unaffected by growth restriction where bloods were collected around the same gestational ages. **** p<0.0001. Error bars are mean ± SEM.

 **Supplementary Figure S2:** Expression of five circulating mRNAs among 16 women who had bloods taken just prior to (Pre), and 24 hours after (Post) an intramuscular injection of corticosteroid (11.4 mg of betamethasone). These are the same five mRNAs as those shown in Fig. 1 of the main manuscript. None were significantly different after the injection of corticosteroids. Error bars are mean ± SEM.

**Supplementary Figure S3:** Expression of eight circulating mRNAs among 16 women who had bloods taken just prior to (Pre), and 24 hours after (Post) an intramuscular injection of corticosteroid (11.4 mg of betamethasone). These are the same eight mRNAs as those shown in supplementary Fig. S1. ** p<0.01, *** p<0.001, **** p<0.0001. Error bars are mean ± SEM.

****

**A**

**B**

**C**

**D**

**E**

**Supplementary Figure 4**

****

****

**

****

***

****

***

****

**

****

**Supplementary Figure S4:** Expression of the five circulating mRNAs in the FOX cohort where the cases of preterm fetal growth restriction were split according to whether there was co-existent preeclampsia, or not.

FGR – Preterm fetal growth restriction only (n=65).

FGR + preeclampsia – Presence of preterm fetal growth restriction with co-existent preeclampsia (n=63).

Comparisons of these two groups were vs controls, which were ongoing pregnancies where bloods were taken around the same gestation as cases.

Kruskal-Wallis analysis performed. ** p<0.01, *** p<0.001, **** p<0.0001. Error bars are mean ± SEM.
